# Effectiveness of an on-site vaccination campaign for patients with cancer

**DOI:** 10.1186/s12889-026-29157-4

**Published:** 2026-09-07

**Authors:** Theresa Markus, Sarah Eitze, Ruth Flümann, Philipp Gödel, Michael Hallek, Sebastian Herrmann, Udo Holtick, Jonathan Köllges, Petra Langerbeins, Janina Leckler, Clara Lehmann, Nathalie Lehnen, Ben Mechtel, Johanna Prinz, Tim Richardson, Jan Rybniker, Jon Salmanton-García, Rosanne Sprute, Jonathan Steinke, Jannik Stemler, Thomas Zander, Oliver A. Cornely, Sibylle C. Mellinghoff

**Affiliations:** 1 Institute of Translational Research, Cologne Excellence Cluster On Cellular Stress Responses in Aging-Associated Diseases (CECAD), Faculty of Medicine and University Hospital Cologne, University of Cologne, Cologne, Germany; 2 Faculty of Medicine and University Hospital Cologne, Department I of Internal Medicine, University of Cologne and Centre for Integrated Oncology Aachen Bonn Cologne Duesseldorf (CIO ABCD) and Excellence Centre for Medical Mycology (ECMM), University of Cologne, Cologne, Germany; 3 Institute for Planetary Health Behaviour, University of Erfurt, Erfurt, Germany; 4 German Centre for Infection Research (DZIF), Partner Site Cologne/Bonn, Cologne, Germany; 5 University of Cologne, Faculty of Medicine and University Hospital Cologne, Clinical Trials Centre Cologne (ZKS Köln), Cologne, Germany; 6 Department I for Internal Medicine, Kerpener Str. 62, Cologne, 50937 Germany

**Keywords:** Vaccination rates, Haematology and oncology, Vaccine campaign, Influenza, COVID-19, Pneumococcal, Herpes zoster

## Abstract

**Background:**

Patients with haematological or oncological malignancies are at increased risk of severe infections. Vaccination is a key preventive strategy and is recommended by National Immunization Technical Advisory Groups (NITAGs). Yet, vaccination rates in this population remain improvable. This project aimed to evaluate and enhance vaccine uptake in cancer patients.

**Methods:**

We conducted a pre- and post-observational study at the University Hospital Cologne in Germany to assess vaccination status over a 1.5-year intervention. Relevant infections included COVID-19, influenza, herpes zoster, pneumococci and RSV. The campaign included patient education, staff training, on-site vaccination, and targeted promotional efforts.

**Results:**

A total of 749 patients were included (n = 398 in 2023, n = 351 in 2025). Overall vaccination rates increased from 43 to 49%, while COVID-19 vaccine coverage remained above 90%. Notable increases were observed for influenza (37% to 48%), pneumococcal (16% to 36%), and herpes zoster (22% to 33%) vaccines. The proportion of patients fully vaccinated according to public health recommendations rose from 6 to 16%, whereas those completely unvaccinated remained low (4% to 6%).

**Conclusion:**

Focused vaccination campaigns can effectively increase vaccine uptake in patients with cancer; however, the proportion of individuals vaccinated per recommended schedule remains low. Future research should investigate barriers and reasons for vaccine refusal to guide more targeted interventions.

**Supplementary Information:**

The online version contains supplementary material available at https://doi.org/10.1186/s12889-026-29157-4.

## Introduction

Patients with haematological or oncological malignancies are at high risk of infection, severe disease and higher mortality. This risk includes vaccine-preventable infections emphasizing the importance of vaccination in clinical care for these patients [1–3]. Vaccine-induced humoral and cellular immune response is often impaired by the underlying disease as well as immunosuppressive treatment [4, 5].Consistent and comprehensive vaccine administration is essential to protect high-risk populations, alongside research-driven optimization of vaccination schedules [6].

The Standing Committee on Vaccination (STIKO) and the Infectious Diseases Working Party for Infections in Haematology and Oncology (AGIHO) of the German Society for Haematology and Medical Oncology (DGHO) recommend routine vaccination against coronavirus diseases 2019 (COVID-19), influenza, pneumococcal disease, herpes zoster, and respiratory syncytial virus (RSV) for immunocompromised individuals [1, 7]. Despite these recommendations, vaccination rates in Germany remain alarmingly low. According to the national public health institute, the Robert Koch Institute (RKI), only 34.0% of adults with underlying conditions were vaccinated against influenza during the 2024/2025 season, and pneumococcal vaccination coverage reached only 24.0% by early 2024 [8]. First-dose coverage of herpes zoster vaccination among individuals over 60 years was as low as 11.5%, and second-dose coverage at 7.7% in 2022 [9].

Finally, the COVID-19 pandemic underscored the importance of robust vaccination efforts while also influencing public perception, both raising awareness of vaccine benefits and contributing to increased hesitancy [10]. To improve vaccine coverage among patients with haematological or oncological malignancies, the University Hospital Cologne (UHC) in Germany launched a vaccination campaign incorporating patient education, staff training, practical on-site implementation, and targeted awareness campaigns. Here, we describe the strategies employed and report pre- and post-intervention vaccination rates.

## Materials and methods

### Study setting

This cohort study retrospectively analysed vaccination histories of inpatients treated at the Department of Internal Medicine I at the Cologne University Hospital, a university institution providing maximum care and specialized in hematology, oncology, clinical infectious diseases, immunology, and internal intensive care medicine. All patients admitted during the study periods were included whenever vaccination history was available. Vaccination coverage was assessed at two predefined time points: first during the baseline assessment from August to October 2023 and subsequently during the post-intervention assessment from February to April 2025. Between these assessments, a multi-component vaccination campaign was implemented from October 2023 to January 2025. The campaign included recurring educational initiatives for healthcare staff and patients, dissemination of informational material, targeted outreach activities, and an on-site vaccination week conducted from October 7–11, 2024. RSV vaccination was incorporated into the campaign following the introduction of the vaccine recommendation in Germany in autumn 2024.

### Patient population

Adult patients ≥ 18 years with haematological or oncological disease were included in this study. Only complete primary immunizations for the above-mentioned vaccines were recorded. A retrospective chart review considering vaccination history, sex and age was conducted. For subgroup analyses, patients were stratified into five birth-year cohorts (1928–1945, 1946–1964, 1965–1980, 1981–1996, and 1997–2010). These cohorts were not designed to represent equally sized intervals but were chosen to reflect clinically and epidemiologically relevant age groups with differing vaccination recommendations, risk profiles, and expected vaccination behaviours. To improve readability, the cohorts were consistently referred to throughout the manuscript using descriptive labels (‘oldest cohort’, ‘older cohort’, ‘middle-aged cohort’, ‘younger cohort’, and ‘youngest cohort’).

### Data capture

Data collection included vaccination status documented electronically and patient-specific variables, including type of haematological or oncological disease, age group, and sex (male, female, diverse). Vaccination data included status regardless of the product used. For COVID-19 vaccines, the data included basic immunizations (one or two doses, depending on the specific vaccine) as well as first and second booster doses. Influenza vaccination status was determined based on administration during the 2022/23 or 2024/25 seasons. This was referred to as the *current season*. Influenza vaccination status was assessed using two separate variables: ‘Seasonal influenza vaccination’ referred to vaccination received during the respective current influenza season (2022/23 and 2024/25), whereas ‘regular annual influenza vaccination’ referred to repeated influenza vaccination behaviour across previous seasons, including at least the immediately preceding season. Depending on the vaccine, individuals are considered fully vaccinated after completing the recommended vaccination schedule: a single dose for standard pneumococcal vaccination, two doses for the recombinant herpes zoster vaccine, and a single dose for RSV. With regard to influenza, a patient was considered “vaccinated” if they had been vaccinated against influenza in the previous season. “Complete” vaccination included vaccination against COVID-19, influenza, pneumococci, and for patients above the age of 50 Herpes Zoster. From 2025 this included RSV vaccination for patients aged 60 years or more, too. All patient-related information was collected in an anonymised format. No systematic assessment of reasons for vaccine refusal or non-vaccination was performed as part of the study protocol. Observations regarding potential barriers to vaccination emerged only during informal patient counselling interactions and were therefore considered anecdotal qualitative impressions rather than systematically collected study data.

### Concept of the vaccination campaign between 2023 and 2025

The vaccination campaign aimed to improve awareness and vaccination rates among medical staff and patients through a series of targeted initiatives. These efforts were designed to provide comprehensive education, facilitate vaccination access, and ensure consistent communication of vaccination guidelines.

#### Educational initiatives for medical staff

Regular training sessions took place during the weekly clinic meetings and were supplemented by specialised sessions for resident physicians. The sessions provided current data on vaccination recommendations from STIKO/RKI and medical societies. Also, tailored teaching sessions on vaccination standards were held at the different wards at the clinic.

**Fig. 1:**
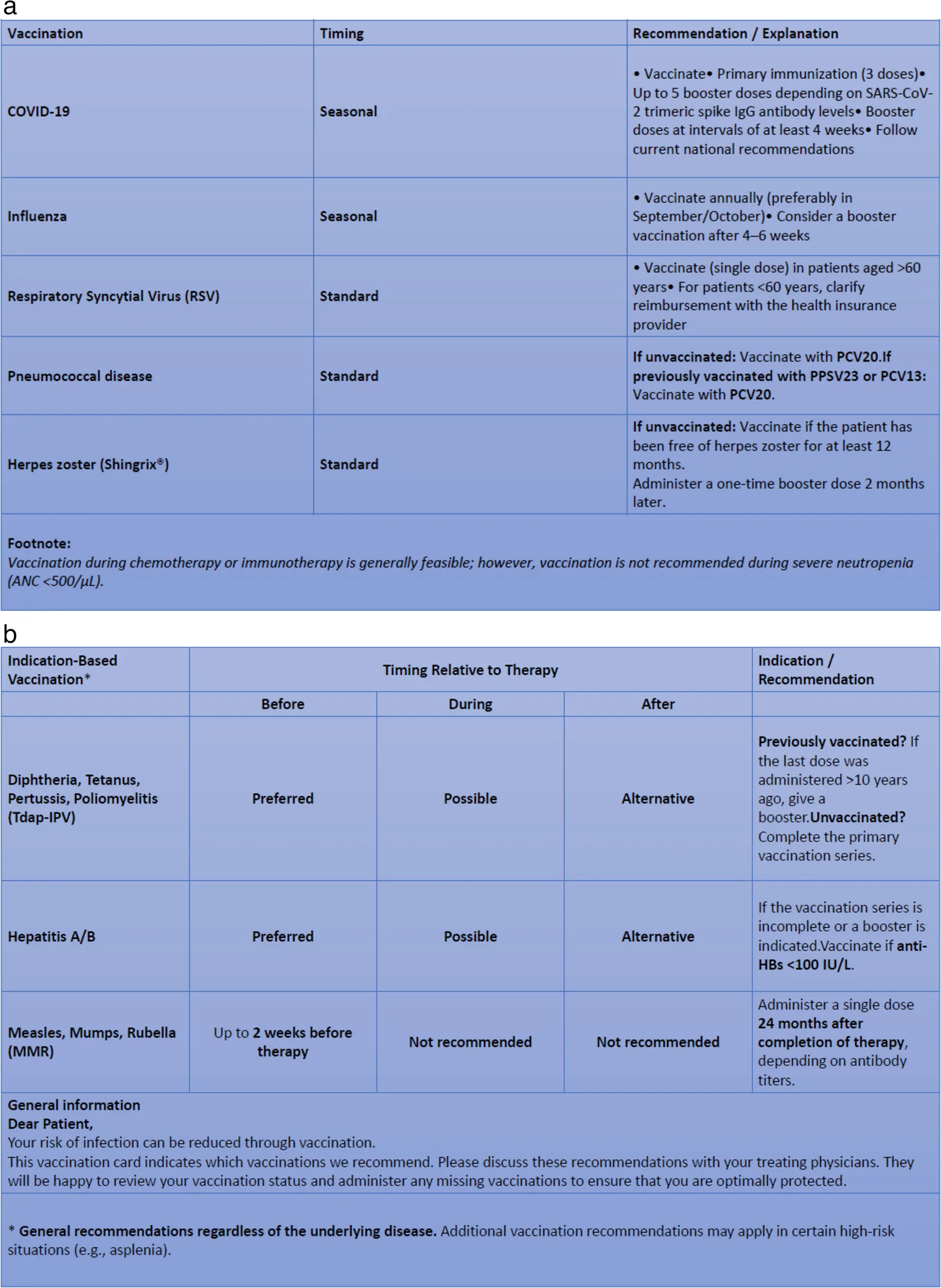
Vaccination Pocket Card. Pocket card summarizing evidence-based vaccination recommendations for patients undergoing chemotherapy or immunotherapy and (**A** and **B**). The card provides guidance on the timing of vaccines relative to treatment cycles, and highlights vaccines indicated for routine and high-risk immunization. It is intended as a quick-reference tool for clinicians to optimize immunization strategies in immunocompromised patients

Moreover, a specialised task force was formed to promote vaccination across different subdivisions of the department including physicians from the haematology and oncology wards, the stem cell transplantation out-patient clinic, and the general haematology and oncology outpatient clinic, as well as infectious disease specialists. This team created pocket cards that summarised the most recent vaccination recommendations (Fig. 1A and B; original version in German can be found in Supplement) and disseminated them.

#### Patient-centred efforts

Both online and face-to-face educational opportunities were offered regularly to patients. Online training courses were offered, and an information session took place during the Centre for Integrated Oncology (CIO) Cologne’s online patient day on October 21, 2024 – an initiative for patient information that regularly (monthly) takes place and addresses various topics around cancer medicine. A total of 62 patients attended a presentation titled “Vaccination Protection for Cancer Patients – What Needs to Be Considered.” An e-mail address (Cio-impfen@uk-koeln.de) was provided to address any further questions. Moreover, a vaccination week was organized from October 7 to 11, 2024, which included an information booth at the CIO outpatient clinic. Patients were provided with customized information regarding the advantages and potential adverse effects of vaccination, as well as flyers, vaccination pocket cards, and the chance to receive seasonal vaccines. Additionally, the programme provided certain patients with the chance to have their RSV vaccination titres tested.

#### Community and public awareness

Community and Public Awareness: Outreach initiatives focused on medical practices by sending flyers and emails to practitioners of general and internal medicine and by incorporating five additional training sessions into educational programmes for resident physicians.

#### Collaboration with other projects

The campaign intersected with other initiatives aimed at improving immunisation protection for cancer patients. Notably, an RSV study was integrated into the vaccination week, offering patients additional opportunities for testing of vaccine-induced immunity.

### Statistical analysis

**Fig. 2:**
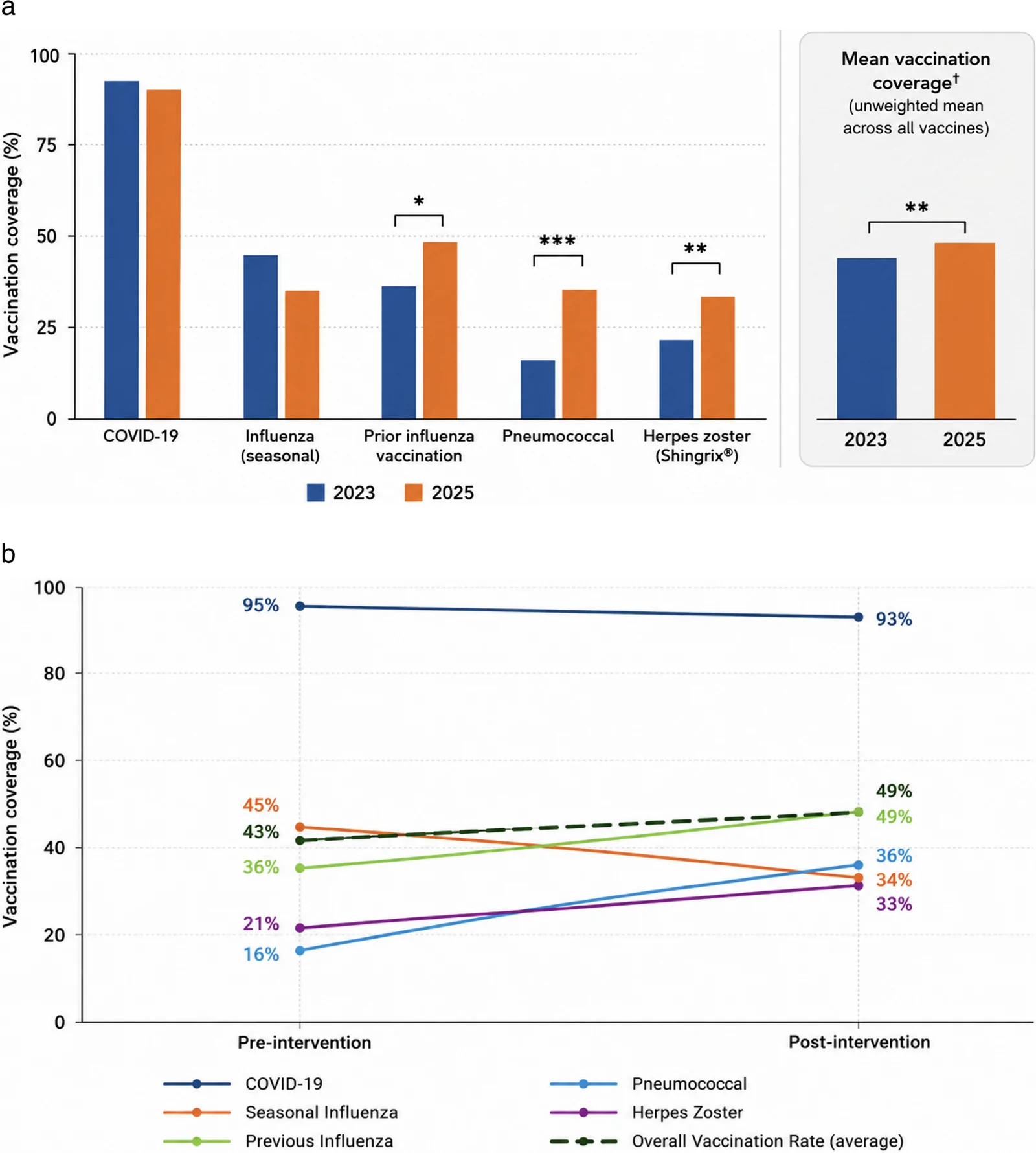
Vaccination coverage by vaccine. Bar chart (**A**) and graph (**B**) showing vaccination coverage for individual vaccines in the years 2023 and 2025. Statistical significance for changes between years is indicated as p < 0.05, p < 0.01, and p < 0.001. The final bar represents the unweighted mean coverage across all vaccines; no formal statistical test was performed for this summary measure

Statistical analysis was performed using IBM SPSS Statistics (version 29.0.2.0; IBM Corp., Armonk, NY, USA). Vaccination rates between 2023 and 2025 were compared using cross-tabulations and Pearson’s chi-square tests, as the study samples were independent and the variables categorical. All statistical tests were two-sided, and a p-value < 0.05 was considered statistically significant. Beyond vaccine-specific analyses, an overall descriptive summary measure of vaccination coverage was calculated as the unweighted mean across all evaluated vaccines. An unweighted approach was chosen because eligibility criteria and denominators differed substantially between vaccines, particularly due to age-dependent recommendations for herpes zoster and RSV vaccination. Consequently, this summary measure was intended for descriptive purposes only, and no inferential statistical comparison was performed for the overall mean vaccination coverage (Fig. 2a).

### Data security and ethics approval

All data used to assess vaccination status were anonymised prior to analysis. Additionally, ethical approval for the study was obtained (No. 25–1242).

## Results

Vaccination rates were assessed before and after an intensified vaccination campaign in 2023 (n = 398) and 2025 (n = 351) depicted in Table 1. Patient characteristics are depicted in Table 2. A total of 201 women and 197 men were included in the 2023 campaign, compared to 194 women and 157 men in the 2025 campaign. The main text reports the most notable findings, while the supplement (Supp. Table 1 and Suppl. Figure 1–3) presents the full results. The mean vaccination coverage across all vaccines increased from 42.9% in 2023 to 49.1% in 2025 (Fig. 2a and b). In our vaccination week a total of 38 patients were vaccinated: 17 received the influenza vaccine, 11 the COVID-19 vaccine, and 11 the RSV vaccine.

**Table 1:** Descriptive results of vaccination rates before and after the intervention

	**2023 (n = 398)**		**2025 (n = 351)**		
Vaccination	Yes n (%)	No n (%)	Yes n (%)	No n (%)	*P*-value^*^ (2023 vs. 2025)
COVID-19	377 (94.7)	21 (5.3)	327 (93.2)	24 (6.8)	0.370
Seasonal Influenza	177 (44.5)	221 (55.5)	120 (34.2)	231 (65.8)	0.004
Previous Influenza	146 (36.7)	252 (63.3)	170 (48.4)	181 (51.6)	0.001
Pneumococcal	65 (16.3)	333 (83.7)	127 (36.2)	224 (63.8)	< 0.001
Herpes zoster	88 (22.1)	310 (77.9)	117 (33.3)	234 (66.7)	< 0.001

^*^Pearson`s chi-square test

**Table 2:** Vaccination rates 2023 and 2025 in relation to age and sex

			COVID-19		Seasonal Influenza		Previous Influenza		Pneumococcal		Herpes Zoster	
Birth Cohort	Survey		Vaccinated (n,%)	Not Vaccinated (n,%)	Vaccinated (n,%)	Not Vaccinated (n,%)	Vaccinated (n,%)	Not Vaccinated (n,%)	Vaccinated (n,%)	Not Vaccinated (n,%)	Vaccinated (n,%)	Not Vaccinated (n,%)
oldest cohort	2023	N = 64	58 (90.6)	6 (9.4)	31 (48.4)	33 (51.6)	36 (56.3)	28 (43.8)	24 (37.5)	40 (62.5)	34 (53.1)	30(46.9)
	2025	N = 10	10 (100)	0 (0)	4 (40.0)	6 (60.0)	7 (70.0)	3 (30.0)	5 (50.0)	5 (50.0)	4 (40.0)	6 (60.0)
Older cohort	2023	N = 131	127 (96.9)	4 (3.1)	52 (39.7)	79 (60.3)	48 (36.6)	83 (63.4)	19 (14.5)	112 (85.5)	19 (14.5)	112 (85.5)
	2025	N = 180	167 (91.6)	11 (8.4)	72 (40.0)	108 (60.0)	100 (55.6)	80 (44.4)	77 (42.8)	103 (57.2)	81 (45.0)	99 (55.0)
middle-aged cohort	2023	N = 103	98 (95.1)	5 (4.9)	44 (42.7)	59 (57.3)	37 (35.9)	66 (64.1)	13 (12.6)	90 (87.4)	18 (17.5)	85 (82.5)
	2025	N = 95	91 (95.8)	4 (4.2)	29 (30.5)	66 (69.5)	43 (45.2)	52 (54.7)	24 (25.3)	71 (74.7)	20 (21.1)	75 (78.9)
younger cohort	2023	N = 87	82 (94.3)	5 (5.7)	43 (49.4)	44 (50.6)	21(24.1)	66 (75.9)	8 (9.2)	79 (90.8)	16 (18.4)	71 (81.6)
	2025	N = 40	36 (90.0)	4 (10.0)	8 (20.0)	32(80.0)	10 (25.0)	30 (75.0)	10 (25.0)	30 (75.0)	9 (22.5)	31 (77.5)
youngest cohort	2023	N = 13	12 (92.3)	1(7.7)	7(53.8)	6 (46.2)	4 (30.8)	9 (69.2)	1 (7.7)	12 (92.3)	1 (7.7)	12 (92.3)
	2025	N = 26	23 (88.5)	3 (11.5)	7 (26.9)	19 (73.1)	10 (38.5)	16 (61.5)	11 (42.3)	15 (57.7)	3 (11.5)	23 (88.5)
Sex												
Male	2023	n = 197	188 (95.4)	9 (4.6)	93 (47.2)	104 (52.8)	81 (41.1)	116 (58.9)	32 (16.2)	165 (83.8)	31 (15.7)	166 (84.3)
	2025	n = 157	147 (93.6)	10 (6.4)	57 (36.3)	100 (63.7)	84 (53.5)	73 (46.5)	56 (35.7)	101 (64.3)	52 (33.1)	105 (66.9)
Female	2023	n = 201	189 (94)	12 (6)	84 (41.8)	117 (58.2)	65 (32.3)	136 (67.7)	33 (16.4)	168 (83.6)	57 (28.4)	144 (71.6)
	2025	n = 194	180 (92.8)	14 (7.2)	63 (32.5)	131 (67.5)	86 (44.3)	108 (55.7)	71 (36.6)	123 (63.8)	65 (33.5)	129 (66.5)

### COVID-19 vaccination

There was a non-significant (p = 0.370) decrease in COVID-19 vaccination coverage from 94.7% to 93.2%. This trend was consistent across sex and age groups. Coverage in the youngest cohort declined from 92.3% to 88.5% (p = 0.709). In contrast to the youngest cohort, the oldest cohort reached a vaccination coverage of 100% in 2025 (p = 0.031).

### Regular annual influenza vaccination

The percentage of patients receiving regular annual influenza vaccination increased in all age groups. There was a statistically significant increase in influenza vaccination coverage following the implementation of the campaign: overall uptake rose from 36.7% to 48.4% (p = 0.001). This positive trend was observed across sexes, although at different levels: among males, coverage increased from 41.1% to 53.5% (p = 0.020), and among females, from 32.3% to 44.3% (p = 0.014). The most pronounced improvement was seen in the older cohort, with vaccination rates rising from 36.6% to 55.6% (p < 0.001).

### Seasonal influenza vaccination

There was a statistically significant decrease in influenza vaccination coverage specifically for the current influenza season, 2025, from 44.5% to 34.2% (p = 0.004). This reduction was observed across all cohorts; however, subgroup analyses revealed important trends. A statistically significant decline among males was noted, with coverage decreasing from 47.2% to 36.3% (p = 0.039). Among females, the vaccination coverage rate decreased from 41.8% to 32.5% (p = 0.055). Seasonal influenza vaccination coverage decreased across all age groups, although at different levels. The largest significant decline was observed in the younger cohort, with coverage falling from 49.4% to 20.0% (p = 0.002). While regular annual influenza vaccination behaviour increased following the intervention, vaccination uptake during the current influenza season declined.

### Pneumococcal vaccination

The strongest average increase was observed for pneumococcal vaccination. There was a statistically significant increase in pneumococcal vaccination coverage and overall uptake doubled from 16.3% to 36.2% (p < 0.001).

### Herpes zoster vaccination

There was a statistically significant increase in coverage for the herpes zoster vaccine, with overall uptake rising from 22.1% to 33.3% (p < 0.001). Vaccination coverage showed an increase for both male and female post-intervention, narrowing the gap between sexes. Specifically, the vaccination rate for male rose significantly from 15.7% to 33.1% (p < 0.001).

### RSV

The RSV vaccine has become available for elderly (≥ 75 years of age) and risk groups (≥ 60 years of age) in Germany since autumn 2024 [11]. Out of 351 individuals (2025), 15 were vaccinated (4.3%). Vaccine uptake was equal between sex groups: 4.5% among male and 4.1% among females. Due to the small sample size of 15 vaccinated individuals, no detailed statistical assessments are reported.

### Completely vaccinated and unvaccinated individuals

In 2023, 5.5% (n = 22) of people were completely vaccinated. The percentage increased to 16.2% (n = 57) in 2025. In contrast, 3.8% (n = 15) of patients were completely unvaccinated in the pre-intervention analysis. The percentage of completely unvaccinated patients had increased in the second analysis in 2025 (6.0%, n = 21). During patient counselling throughout the vaccination campaign, several recurring reasons for non-vaccination were identified, including vaccine opposition, incorrect medical advice, insufficient awareness, concerns related to the underlying disease, and organisational or time-management barriers. As these observations were not systematically documented within the study design, they should be interpreted as qualitative exploratory findings.

## Discussion

Vaccination is crucial for preventing severe infections, consecutive hospitalisation and death in patients with haematological and oncological diseases [1, 12, 13]. Our multi-componential vaccination campaign has resulted in an increase of vaccination rates in these patients.

The vaccination campaign did not result in a notable increase in COVID-19 vaccination uptake. It may be assumed that, due to the pandemic and the associated heightened public attention, individuals had already been exposed to a range of effective interventions, thereby largely exhausting the potential for further improvement before our intervention (average vaccination coverage rate above 88%). The lowest vaccination coverage was seen in the youngest cohort with a rate of 88.5%. National data for the 2023/24 winter season similarly show lower coverage in healthy adults aged 18–59 (approx. 50–60%) compared to those aged ≥ 60 (approx. 75%) [14]. Due to evidence from several different cohorts of haematological as well as oncological patients, a repeated booster vaccination should be recommended to all those patients that undergo immunosuppression [15].

The study revealed shortcomings in seasonal influenza vaccination coverage within the 2025 season. This observation is consistent with national surveillance data from the STIKO/RKI vaccination monitoring reports, which demonstrated that influenza vaccination rates in Germany had already shown a declining trend before the COVID-19 pandemic, followed by a temporary increase during the pandemic period and a subsequent renewed decline thereafter [16]. A similar trend has been observed by a US study recently published. Whether this decline is a seasonal phenomenon or a general trend needs to be thoroughly observed. A follow-up study assessing the reasons for vaccine hesitancy will further investigate this evolution. However, our results suggest that educational measures alone may be insufficient for maintaining high vaccination coverage in vulnerable groups. Structural interventions are needed to integrate vaccination into existing care pathways and improved communication strategies [17] to reach vaccine hesitant patients. To address structural barriers, vaccinations should be accessible across all care settings including the inpatients setting. The distinction between regular annual influenza vaccination behaviour and vaccination uptake during the current influenza season suggests that long-term acceptance of influenza vaccination may have improved, whereas season-specific uptake remained influenced by broader temporal and societal trends.

The most significant vaccination success was achieved for pneumococcal vaccination. Given the increased risk for severe disease by invasive pneumococcal infections in immunocompromised individuals, pneumococcal vaccination is strongly recommended for patients with lymphoma to prevent pneumococcal infections [18, 19]. According to the RKI, only 20% of people aged ≥ 60 years and 20% of adults with a relevant underlying disease are vaccinated against pneumococci [14, 20]. Although the pre-intervention cohort was initially below this value, the post-intervention cohort exceeded it by 16%. Our results are consistent with results from another German multicentre intervention which implemented a focused vaccination strategy (Easy Vaccination in Oncology [EVO]) and reports increased rates for different vaccinations (herpes zoster, hepatitis B, influenza, and pneumococci) in the at-risk population of oncological patients [21]. Various studies show that medical recommendations are one of the strongest predictors of willingness to be vaccinated [22]. In the case of pneumococcal vaccination we therefore hypothesize that lacking recommendation is the main driver of under-vaccination as assumed previously [23]. Importantly, the updated STIKO recommendation introduced in September 2023 supporting the use of PCV20 may have further contributed to the observed increase in vaccination rates [24]. The simplified vaccination regimen with PCV20, compared with the previously more complex sequential strategies involving PCV13 and PPSV23, likely facilitated implementation in clinical practice. This underscores that vaccination recommendations must be clear and straightforward to ensure widespread uptake.

The herpes zoster vaccination also showed a significant increase within the frame of the campaign. The recombinant zoster vaccine has been approved in patients 50 years due to its high capability to prevent symptomatic herpes zoster reactivation and is strongly recommended for cancer patients [25]. Our study results are consistent with previous research showing that vaccination rates in the target population of immunocompromised individuals have increased only modestly over time [26]. Organisational and educational interventions proved successful in increasing the vaccination rates further in this study. Improved education during outpatient treatment and the COVID-19 pandemic were considered contributing factors. While the RKI had also reported a significant increase in herpes zoster vaccination since 2018, we were similarly able to improve the coverage in our patients. According to the RKI, 18 percent of people aged ≥ 50 years with an underlying condition are vaccinated against herpes zoster [27]. However, concerns about potential side effects may contribute to persistent hesitancy and lower uptake in this population [28].

We found that older age groups have higher vaccination rates compared to younger groups. This observation is consistent with findings from the RKI VacMap analyses, which similarly demonstrated increasing vaccination uptake with higher age among adults at risk due to comorbidities and chronic underlying diseases [29]. Also, an Austrian study on influenza vaccination found that older age correlated with greater willingness to vaccinate [30]. This highlights the need to target younger groups in vaccination programs and promote further research into reasons for vaccine hesitancy among those; especially the role of information spread by social media needs to be examined in future [31]. Importantly, younger immunocompromised patients still face a substantial risk of severe infectious complications and thus have a clear need for vaccination. While other studies have reported stronger correlations with education or gender rather than age, our results indicate a persistent age-related gap. However, educational status was not assessed, which could be a potential confounder [32]. Future investigations should incorporate sociodemographic and behavioural factors to better understand and address these disparities.

Our study also provides one of the first assessments of RSV vaccination uptake following the introduction of RSV vaccination recommendations in Germany. Despite being one of the primary target populations, vaccination coverage remained low approximately one year after implementation. Although our study was not designed to investigate the reasons underlying this finding, the results suggest that introducing a new vaccination programme alone may be insufficient to achieve broad uptake among patients with cancer. Additional structural, communicative, and educational measures to facilitate informed vaccination decisions and improve access may therefore be required. Future studies should investigate the barriers and facilitators influencing RSV vaccine uptake in this vulnerable population.

Several limitations should be considered when interpreting our findings. First, the relatively small sample size and the unequal distribution of participants across study groups may have limited statistical power for some subgroup analyses. Second, the uncontrolled pre–post design without a contemporaneous control group precludes causal inference. Consequently, although vaccination coverage improved following implementation of the intervention, these changes cannot be attributed solely to the intervention itself, as secular trends, increasing public awareness, evolving national recommendations, or other external factors may also have contributed. Furthermore, because the intervention comprised several components—including educational sessions, pocket vaccination cards, physician reminders, and an on-site vaccination campaign—the individual contribution of each component cannot be determined. Third, the already high baseline COVID-19 vaccination coverage observed in our cohort suggests a ceiling effect, consistent with findings from the EVO study, thereby limiting the potential for further improvement in this vaccine category [21]. Fourth, potential self-selection bias and the single-centre setting at a university hospital may limit the generalisability of our findings to other patient populations and healthcare settings. Finally, reasons for vaccine acceptance or non-vaccination were not systematically assessed. Consequently, the present study cannot determine which factors influenced vaccination decisions, nor can it assess participants' motivation, engagement, openness towards vaccination, or perceptions of the different intervention components. A follow-up study is planned to investigate these determinants in greater detail.

The educational activities, informational materials, and on-site vaccination campaign were implemented successfully within routine clinical care and were accompanied by improvements in vaccination coverage across several vaccine categories. Although participation in the offered activities and the demand for practical resources such as vaccination pocket cards suggest that these measures were feasible and well accepted in our institution, formal implementation outcomes—including participant satisfaction, engagement, or behavioural mechanisms—were not assessed. Future studies incorporating these measures will be important to identify which implementation strategies are most effective and to optimize vaccination programmes for patients with cancer.

Overall, the intervention demonstrated that targeted educational and organisational measures can contribute to improved vaccination uptake in patients with cancer. Nevertheless, the relatively low proportion of completely vaccinated individuals after the intervention indicates that educational campaigns alone are unlikely to fully close existing vaccination gaps and should be complemented by broader structural implementation strategies.

Our initiative achieved broad outreach, with high patient participation in educational sessions reflecting strong engagement and receptiveness to vaccine-related information. These findings suggest that combining patient education with accessible, tailored informational resources can support enhancing vaccine uptake among immunocompromised patients, but education alone is not enough to achieve optimal coverage. Future efforts should focus on identifying persistent barriers to vaccination, strengthening and standardizing educational strategies, promoting combination vaccine use, and implementing flexible on-site vaccination services to address the persistently low coverage rates and further optimize protection. Such investigations may benefit from applying the validated 7-C model to systematically assess vaccination willingness and improve comparability across studies.

## Supplementary Information

Supplementary Material 1: Supplemental Fig. 1. Comparison of vaccinated patients by sex in 2023 and 2025. Graph illustrating the proportion of vaccinated male and female patients in the years 2023 and 2025. Differences between sexes and across years are shown. Supplemental Fig. 2. Comparison of vaccinated patients by age group in 2023 and 2025. Graph illustrating the proportion of vaccinated age groups in the years 2023 and 2025. Differences between age groups and across years are shown. Supplemental Fig. 3. Comparison of vaccinated patients by underlying disease in 2023 and 2025. Graph illustrating the proportion of vaccinated malignancy groups in the years 2023 and 2025. Differences between malignancy groups and across years are shown. Supplemental Fig. 4a. Vaccination Pocket Card 1a in original German Version. Supplemental Fig. 4b. Vaccination Pocket Card 1b in original German Version. Supplemental Table 1.

## Data Availability

The data that support the findings of this study are available from the corresponding author upon reasonable request.

## Abbreviations

AGIHO: Infectious Diseases Working Party of the German Society for Haematology and Medical Oncology
CIO: Centre for Integrated Oncology
COVID-19: Coronavirus disease 2019
DGHO: German Society for Haematology and Medical Oncology
EVO: Easy Vaccination in Oncology
NITAG: National Immunization Technical Advisory Group
PCV13: 13-Valent pneumococcal conjugate vaccine
PCV20: 20-Valent pneumococcal conjugate vaccine
PPSV23: 23-Valent pneumococcal polysaccharide vaccine
RKI: Robert Koch Institute
RSV: Respiratory syncytial virus
SPSS: Statistical Package for the Social Sciences
STIKO: Standing Committee on Vaccination
UHC: University Hospital Cologne
ZKS: Clinical Trials Centre (Zentrum für Klinische Studien)
