# Supplementary material for: Effectiveness of an on-site vaccination campaign for patients with cancer
Source: BMC Public Health. 2026 Sep 7;26:2540. doi: 10.1186/s12889-026-29157-4 (PMC13551785; doi:10.1186/s12889-026-29157-4)
Supplement: Supplementary file 1 [file 12889_2026_29157_MOESM1_ESM.docx]

**Supplement**

**Supplemental Figure 1. Comparison of vaccinated patients by sex in 2023 and 2025.**

**Supplemental Figure 2. Comparison of vaccinated patients by age groups in 2023 and 2025.**

**Supplemental Figure 3. Comparison of vaccinated patients by underlying disease in 2023 and 2025.**

**Supplemental Figure 4a. Pocket Card. Pocket Card with vaccination recommendations for patients undergoing chemo-/immunotherapy. Original version in German.**

**Supplemental Figure 4b Pocket Card. Pocket Card with vaccination recommendation for patients undergoing chemo-/immunotherapy. Original version in German.**

 **Supplemental Table 1** Vaccination rates 2023 and 2025 in relation to the underlying malignancy
